# Extensively Drug-Resistant *Mycobacterium tuberculosis* from Aspirates, Rural South Africa

**DOI:** 10.3201/eid1603.091486

**Published:** 2010-03

**Authors:** Scott K. Heysell, Anthony P. Moll, Neel R. Gandhi, François J. Eksteen, Palav Babaria, Yacoob Coovadia, Lynn Roux, Umesh Lalloo, Gerald Friedland, N. Sarita Shah

**Affiliations:** Tugela Ferry Care and Research Collaboration, Tugela Ferry, South Africa (S.K. Heysell, A.P. Moll, N.R. Gandhi, F.J. Eksteen, P. Babaria, U. Lalloo, G. Friedland, N.S. Shah); University of Virginia, Charlottesville, Virginia, USA (S.K. Heysell); Philanjalo Care Center, Tugela Ferry (A.P. Moll, FJ. Eksteen); Yale University, New Haven, Connecticut, USA (P. Babaria, G. Friedland); Albert Einstein College of Medicine and Montefiore Medical Center, Bronx, New York, USA (N.R. Gandhi, N.S. Shah); National Health Laboratory Services, Durban, South Africa (Y. Coovadia, L. Roux); Enhancing Care Initiative-KZN, Nelson R. Mandela School of Medicine, Durban (U. Lalloo)

**Keywords:** Extensively drug-resistant tuberculosis, HIV, lymphadenitis, tuberculous, pleurisy, tuberculous, bacteria, South Africa, CME, dispatch

## CME ACTIVITY

Medscape, LLC is pleased to provide online continuing medical education (CME) for this journal article, allowing clinicians the opportunity to earn CME credit. This activity has been planned and implemented in accordance with the Essential Areas and policies of the Accreditation Council for Continuing Medical Education through the joint sponsorship of Medscape, LLC and Emerging Infectious Diseases. Medscape, LLC is accredited by the ACCME to provide continuing medical education for physicians. Medscape, LLC designates this educational activity for a maximum of 0.25 *AMA PRA Category 1 Credits*™. Physicians should only claim credit commensurate with the extent of their participation in the activity. All other clinicians completing this activity will be issued a certificate of participation. To participate in this journal CME activity: (1) review the learning objectives and author disclosures; (2) study the education content; (3) take the post-test and/or complete the evaluation at www.medscapecme.com/journal/eid; (4) view/print certificate.

## Learning Objectives

Upon completion of this activity, participants will be able to:

- Recognize how concomitant HIV infection can affect the diagnosis of tuberculosis
- Describe procedures and patient characteristics in the current study
- Identify how aspirate cultures can help identify extensively drug-resistant tuberculosis
- Specify the percentage of patients with tuberculosis who were diagnosed with aspirate but not sputum cultures.

## Editor

**Karen L. Foster**, Writer/Editor, Emerging Infectious Diseases. *Disclosure: Karen L. Foster has disclosed no relevant financial relationships.*

## CME AUTHOR

**Charles P. Vega, MD**, Associate Professor; Residency Director, Department of Family Medicine, University of California, Irvine. *Disclosure: Charles P. Vega, MD, has disclosed no relevant financial relationships.*

## AUTHORS

Disclosures: ***Scott K. Heysell, MD, MPH; Anthony P. Moll, MBChB; Neel R. Gandhi, MD; François J. Eksteen, MD; Palav Babaria, MD; Yacoob Coovadia, MBBCh, FCPath; Lynn Roux; Gerald Friedland, MD;*** *and* ***N. Sarita Shah, MD,*** *have disclosed no relevant financial relationships.* ***Umesh Lalloo, MD,*** *has disclosed the following relevant financial relationships: served as an advisor or consultant for AstraZeneca Pharmaceuticals LP, GlaxoSmithKline, and Boehringer Ingelheim Pharmaceuticals, Inc.; served as a speaker or a member of a speakers bureau for AstraZeneca Pharmaceuticals LP, GlaxoSmithKline, Boehringer Ingelheim Pharmaceuticals, Inc.; received grants for clinical research from the National Institutes of Health.*
