# Supplementary material for: Extensively Drug-Resistant Mycobacterium tuberculosis from Aspirates, Rural South Africa
Source: Emerg Infect Dis. 2010 Mar;16(3):557–60. doi: 10.3201/eid1603.091486 (PMC3322040; doi:10.3201/eid1603.091486)
Supplement: Related texts

# Brief report: Extensively Drug-Resistant *Mycobacterium tuberculosis* from Aspirates, Rural South Africa

**DOI:** 10.3201/eid1603.091486

**Authors:** Scott K. Heysell, Anthony P. Moll, Neel R. Gandhi, François J. Eksteen, Palav Babaria, Yacoob Coovadia, Lynn Roux, Umesh Lalloo, Gerald Friedland, N. Sarita Shah

**Affiliations:** Tugela Ferry Care and Research Collaboration, Tugela Ferry, South Africa (S.K. Heysell, A.P. Moll, N.R. Gandhi, F.J. Eksteen, P. Babaria, U. Lalloo, G. Friedland, N.S. Shah); University of Virginia, Charlottesville, Virginia, USA (S.K. Heysell); Philanjalo Care Center, Tugela Ferry (A.P. Moll, FJ. Eksteen); Yale University, New Haven, Connecticut, USA (P. Babaria, G. Friedland); Albert Einstein College of Medicine and Montefiore Medical Center, Bronx, New York, USA (N.R. Gandhi, N.S. Shah); National Health Laboratory Services, Durban, South Africa (Y. Coovadia, L. Roux); Enhancing Care Initiative-KZN, Nelson R. Mandela School of Medicine, Durban (U. Lalloo)

## Abstract

The yield from aspirating lymph nodes and pleural fluid for diagnosing extensively drug-resistant (XDR) tuberculosis is unknown. *Mycobacterium tuberculosis* was cultured from lymph node or pleural fluid aspirates of 21 patients; 7 (33%) cultures grew XDR *M. tuberculosis*. Additive diagnostic yield for XDR *M. tuberculosis* was found in parallel culture of sputum and fluid aspirate.

Tuberculosis (TB) is the leading cause of death among HIV-infected persons in sub-Saharan Africa (*1*). Drug-resistant TB is an emerging public health threat in HIV-prevalent settings, but diagnosis is challenging because of the severely limited laboratory capacity for culture and drug-susceptibility testing (DST). TB diagnosis for HIV-infected patients is particularly challenging because these patients may be more likely to have smear-negative pulmonary disease or extrapulmonary TB (*2*,*3*). Extrapulmonary TB often is diagnosed by clinical findings, indirect measures (e.g., chemistry and cell count of cerebrospinal or pleural fluid, ultrasound of lymph nodes, or pericardial effusions), or smear microscopy for acid-fast bacilli from aspirated extrapulmonary fluid. However, drug-resistant TB is impossible to diagnose by these methods, instead requiring mycobacterial culture and DST (*4*,*5*).

The prevalence of multidrug-resistant and extensively drug-resistant TB (XDR TB) in South Africa has risen exponentially during the past decade. At our rural study site, ≈10% of all TB cases now are drug resistant, and >90% of TB patients are HIV infected (*6*). Death from XDR TB exceeds 80%; most infected persons die before sputum culture and DST results are known (*6*). To improve case detection and decrease diagnostic delay of drug-resistant TB among patients with suspected extrapulmonary TB, we initiated a program to aspirate lymph nodes and pleural fluid for culture and DST. We quantified the yield of these lymph node and pleural fluid aspirates for diagnosing XDR TB.

## The Study

We performed a retrospective cross-sectional study to determine the proportion of patients in whom TB and XDR TB could be diagnosed by culture of fine-needle aspiration of a lymph node or pleural fluid. Additionally, we sought to determine the yield of lymph node and pleural fluid aspiration beyond culture of sputum alone. All patients were eligible who had an aspirate of a noninguinal lymph node or pleural fluid sent for mycobacterial culture and DST during September 1, 2006–December 31, 2008, from the district hospital in rural Tugela Ferry, South Africa. This 355-bed hospital serves 200,000 Zulu persons.

Hospital protocol was for lymph node aspirates to be obtained at the bedside by using sterile technique and large-bore needle at the point of maximal swelling. Pleural fluid was obtained by standard thoracentesis. Care was taken not to introduce air while injecting the fluid specimen into mycobacterial blood culture bottles (BACTEC MycoF-lytic, Becton Dickinson, Sparks, MD, USA). Smear microscopy was not performed on the fluid aspirate. Bottles were transported to the provincial TB referral laboratory in Durban and cultured by using the automated BACTEC 9240 analyzer (Becton Dickinson) in which growth is continuously monitored for 42 days. *Mycobacterium tuberculosis* was confirmed with niacin and nitrate reductase tests. DST of positive cultures was performed by using the 1% proportional method on Middlebrook 7H11 agar for isoniazid (critical concentration, 0.2 µg/mL), rifampin (1 µg/mL), ethambutol (7.5 µg/mL), ofloxacin (2 µg/mL), kanamycin (6 µg/mL), and streptomycin (2 µg/mL) (*7*). XDR TB was defined as *M. tuberculosis* resistant to at least isoniazid, rifampin, ofloxacin, and kanamycin (*8*). Aspirate culture results were compared with sputum culture results if the patient also had a sputum culture performed within 2 weeks of the lymph node or pleural fluid culture. Standard practice was for 1 sputum specimen to be collected for smear microscopy and another specimen to be collected for culture, depending on the patient’s ability to expectorate. Sputum was cultured by using the automated BACTEC MGIT 960 system (Becton Dickinson); DST of positive specimens was performed as described above (*9*).

Medical records were reviewed for basic demographic and clinical data, including age, sex, HIV status, antiretroviral therapy, and TB history. The yield of lymph node and pleural fluid aspirates for detecting *M. tuberculosis* and drug resistance was described by using simple frequencies. Incremental yield of aspirate was calculated for patients who had collection for sputum and either lymph node or pleural fluid aspirate. Ethical approval was obtained from the University of KwaZulu-Natal, Yale University, and Albert Einstein College of Medicine.

For 77 patients, either a lymph node (n = 34) or pleural fluid (n = 33) was aspirated for culture and DST during the study period (Table 1). No patient had both pleural fluid and lymph node aspirates performed.

**Table 1:** Patient characteristics and results of aspirate cultures for *Mycobacterium tuberculosis*, South Africa, September 1, 2006–December 31, 2008*

Characteristic	Lymph node	Pleural fluid
Total no. patients	34	33
Median age, y (IQR)	31 (25–39)	33 (28–39)
Female sex, no. (%)	18 (53)	21 (64)
HIV status, no. (%)		
Positive	30 (88)	24 (73)
Negative	1 (3)	1 (3)
Unknown	3 (9)	8 (24)
Median CD4 cells/mm^3^ (IQR)	128.5† (84–375)	207‡ (118–334)
Receiving ARVs,§ no. (% of HIV-infected)		
Yes	14 (47)	13 (54)
No	13 (43)	10 (42)
Unknown	3 (10)	1 (4)
Median duration on ARVs, wk (IQR)	8.4¶ (2.4–21.1)	10.5# (5.8–55.1)
History of prior TB, no. (%)	8 (24)	9 (27)
Receiving TB treatment,§ no. (%)	20 (59)	18 (55)
Median duration of TB treatment, wk (IQR)	12** (4–16)	6†† (3–14)
Positive culture results, no. (%)		
*M. tuberculosis*	12 (35)	9 (27)
Drug-susceptible TB, no. (% of *M. tuberculosis*)	9 (75)	3 (33)
XDR TB, no. (%)	1 (8)	6 (67)
Missing drug susceptibility results, no. (%)	2 (17)	0
Nontuberculous mycobacteria	1 (3)	0
*Cryptococcus* sp.	0	1 (3)
Other bacteria	2 (6)	1 (3)

*IQR, interquartile range; ARVs, antiretroviral drugs; TB, tuberculosis; XDR TB, extensively drug-resistant TB. †Available for 26 HIV-infected patients, excluding CD4% for 1 infant (36%). ‡Available for 18 HIV-infected patients. §At time of aspirate collection. ¶Available for 10 of 14 patients receiving ARVs. #Available in 12 of 13 patients receiving ARVs. **Available for 9 patients. ††Available for 12 patients.

Of the 34 lymph node cultures performed, 12 (35%) grew *M. tuberculosis*, 1 (3%) grew nontuberculous mycobacteria, and 2 (6%) were other bacteria that were not further speciated (Table 1). Of the 12 positive *M. tuberculosis* cultures, 9 (75%) were drug-susceptible *M. tuberculosis*, and 1 (8%) was XDR *M. tuberculosis*; for 2, DST results were missing. Concurrent sputum samples were available for 6 (50%) of the 12 culture-positive *M. tuberculosis* lymph node aspirates: 3 (50%) were concordant with the aspirate culture (2 drug-susceptible and 1 XDR), and 3 (50%) were sputum culture negative.

Of the 33 pleural fluid cultures performed, 9 (27%) grew *M. tuberculosis*, 1 grew *Cryptococcus* sp., and 1 grew another bacterium (Table 1). Of the 9 *M. tuberculosis* culture-positive pleural fluid specimens, 3 (33%) were drug-susceptible and 6 (67%) were XDR. Among these 9 patients, 5 (55%) had concurrent sputum samples available: 3 (60%) were concordant with the aspirate culture (1 drug-susceptible and 2 XDR), and 2 (40%) were sputum culture negative.

From 17 patients, a sputum sample and either a lymph node or a pleural fluid aspirate was collected for culture and DST (Table 2). For 14, at least 1 specimen was culture positive for *M. tuberculosis*, of which 9 (64%) were positive for sputum, 11 (79%) were positive in the lymph node or pleural fluid, and 5 (36%) were positive by fluid aspirate alone, including 2 patients with XDR *M. tuberculosis* (Table 2).

**Table 2:** Comparison of culture yield for *Mycobacterium tuberculosis* in patients with collection of sputum and either lymph node or pleural fluid aspirate, South Africa, September 1, 2006–December 31, 2008*

Result from sputum	Lymph node, n = 9					Pleural fluid, n = 8			
	DS TB	XDR TB	No DST	No growth		DS TB	XDR TB	No DST	No growth
DS TB	2					1			1
XDR TB		1					2		2
No growth	1		2	3			2		

*DS TB, drug-susceptible tuberculosis; XDR TB, extensively drug-resistant tuberculosis; DST, drug susceptibility testing.

## Conclusions

In this study of predominately HIV-infected patients suspected of having extrapulmonary TB, one third of positive *M. tuberculosis* cultures from lymph node or pleural fluid aspirates were XDR. The additive yield for the diagnosis of any TB of these aspirates above sputum culture alone was 36%. Our findings suggest that strategies of solitary sputum culture or reliance on microscopy of nonsputum fluid analysis would miss opportunities to diagnose drug-resistant TB. Parallel culture of sputum and aspirate fluid appears to be of substantial added benefit for diagnosing XDR TB in this setting.

Our study has several limitations. Aspirates were collected on the basis of the attending physician’s clinical judgment. Therefore, the yield for *M. tuberculosis* and XDR *M. tuberculosis* may be overestimated, and other factors that may have influenced the physician’s suspicion of drug-resistant TB or increased the likelihood of a positive aspirate are not known without additional prospective study. It is also not possible to comment on the true incremental yield after comparing with sputum culture; sputum was not collected from all patients, nor were the reasons for lack of collection documented.

Nonetheless, as co-infection with HIV and TB increases in sub-Saharan Africa, the number of persons with extrapulmonary TB, both drug-susceptible and drug-resistant, is anticipated to rise (*10*,*11*). Therefore, diagnostic algorithms for extrapulmonary TB must consider the critical importance of extrapulmonary fluid culture and DST for diagnosis of drug-resistant TB, particularly in HIV-infected persons. Furthermore, this study highlights the need to validate novel diagnostic tests for *M. tuberculosis* drug resistance on nonsputum fluids.

## References

[1] Corbett EL, Marston B, Churchyard GJ, De Cock KM. Tuberculosis in sub-Saharan Africa: opportunities, challenges and change in the era of antiretroviral therapy. Lancet. 2006;367:926–37. 10.1016/S0140-6736(06)68383-916546541

[2] Shenoi S, Heysell SK, Moll AP, Friedland G. Multi-drug resistant and extensively drug-resistant tuberculosis: consequences for the global HIV community. Curr Opin Infect Dis. 2009;22:11–7. 10.1097/QCO.0b013e328321002019532076PMC3013226

[3] Elliott AM, Namaambof K, Allent BW, Luo N, Hayes RJ, Pobee JO, Negative sputum smear results in HIV-positive patients with pulmonary tuberculosis in Lusaka, Zambia. Int J Tuberc Lung Dis. 1993;74:191–4. 10.1016/0962-8479(93)90010-U8369514

[4] Perenboom RM, Richter C, Swai ABM, Kitinya J, Mtoni I, Chande H, Diagnosis of tuberculous lymphadenitis in an area of HIV infection and limited diagnostic facilities. Trop Geogr Med. 1994;46:288–92.7855914

[5] Hudson CP, Wood R, Maartens G. Diagnosing HIV-associated tuberculosis: reducing costs and diagnostic delay. Int J Tuberc Lung Dis. 2000;4:240–5.10751070

[6] Gandhi NR, Shah NS, Andrews J, Vella V, Moll A, Scott M, Early mortality among MDR and XDR TB patients in rural South Africa. Am J Respir Crit Care Med. 2010;181:80–6. 10.1164/rccm.200907-0989OC19833824

[7] Mitchison DA. Drug resistance in tuberculosis. Eur Respir J Suppl. 2005;25:376–9. 10.1183/09031936.05.0007570415684305

[8] Centers for Disease Control and Prevention. Revised definition of extensively drug-resistant tuberculosis. MMWR Morb Mortal Wkly Rep. 2006;55:1176.

[9] Hanna BA, Ebrahimzadeh A, Elliott LB, Morgan MA, Rusch-Gerdes S, Cio M, Multicenter evaluation of the BACTEC MGIT 960 system for the recovery of mycobacteria. J Clin Microbiol. 1999;37:748–52.998684410.1128/jcm.37.3.748-752.1999PMC84542

[10] World Health Organization. Improving the diagnosis and treatment of smear-negative pulmonary and extrapulmonary tuberculosis among adults and adolescents. Recommendations for HIV-prevalent and resource-constrained settings. Geneva: The Organization; 2006.

[11] World Health Organization. Anti-tuberculosis drug resistance in the world. Report no. 4. Geneva: The Organization; 2008.

# Brief report: Extensively Drug-Resistant *Mycobacterium tuberculosis* from Aspirates, Rural South Africa

**DOI:** 10.3201/eid1603.091486

**Authors:** Scott K. Heysell, Anthony P. Moll, Neel R. Gandhi, François J. Eksteen, Palav Babaria, Yacoob Coovadia, Lynn Roux, Umesh Lalloo, Gerald Friedland, N. Sarita Shah

**Affiliations:** Tugela Ferry Care and Research Collaboration, Tugela Ferry, South Africa (S.K. Heysell, A.P. Moll, N.R. Gandhi, F.J. Eksteen, P. Babaria, U. Lalloo, G. Friedland, N.S. Shah); University of Virginia, Charlottesville, Virginia, USA (S.K. Heysell); Philanjalo Care Center, Tugela Ferry (A.P. Moll, FJ. Eksteen); Yale University, New Haven, Connecticut, USA (P. Babaria, G. Friedland); Albert Einstein College of Medicine and Montefiore Medical Center, Bronx, New York, USA (N.R. Gandhi, N.S. Shah); National Health Laboratory Services, Durban, South Africa (Y. Coovadia, L. Roux); Enhancing Care Initiative-KZN, Nelson R. Mandela School of Medicine, Durban (U. Lalloo)

## Earning CME Credit

To obtain credit, you should first read the journal article. After reading the article, you should be able to answer the following, related, multiple-choice questions. To complete the questions and earn continuing medical education (CME) credit, please go to www.medscapecme.com/journal/eid. Credit cannot be obtained for tests completed on paper, although you may use the worksheet below to keep a record of your answers. You must be a registered user on Medscape.com. If you are not registered on Medscape.com, please click on the New Users: Free Registration link on the left hand side of the website to register. Only one answer is correct for each question. Once you successfully answer all post-test questions you will be able to view and/or print your certificate. For questions regarding the content of this activity, contact the accredited provider, CME@medscape.net. For technical assistance, contact CME@webmd.net. American Medical Association’s Physician’s Recognition Award (AMA PRA) credits are accepted in the US as evidence of participation in CME activities. For further information on this award, please refer to http://www.ama-assn.org/ama/pub/category/2922.html. The AMA has determined that physicians not licensed in the US who participate in this CME activity are eligible for *AMA PRA Category 1 Credits*™. Through agreements that the AMA has made with agencies in some countries, AMA PRA credit is acceptable as evidence of participation in CME activities. If you are not licensed in the US and want to obtain an AMA PRA CME credit, please complete the questions online, print the certificate and present it to your national medical association.

### Article Title: Extensively Drug-Resistant *Mycobacterium tuberculosis* from Aspirates, Rural South Africa

## CME Questions

**1. Which of the following statements about tuberculosis (TB) infection among patients with concomitant HIV infection is most accurate?**

A. Smear-positive pulmonary disease and extrapulmonary TB are more common

B. Smear-positive pulmonary disease is more common, but extrapulmonary TB is less common

C. Smear-negative pulmonary disease and extrapulmonary TB are more common

D. Smear-negative pulmonary disease is more common, but extrapulmonary TB is less common

**2. Which of the following statements about patients participating in the current study is most accurate?**

A. Less than 20% were HIV positive

B. Pleural fluid cultures were much more common than lymph node cultures

C. Most patients had a history of TB

D. Approximately one third of lymph node and pleural fluid cultures grew TB

**3. In what percentage of patients was the positive lymph node or pleural fluid aspirate culture the only source of a culture diagnosis of TB?**

A. 5%

B. 20%

C. 45%

D. 75%

**4. Which of the following statements about the diagnosis of extensively drug-resistant (XDR) TB is most accurate?**

A. More than half of cases of XDR TB were diagnosed by fluid aspirate alone

B. XDR TB was more common in lymph node vs pleural fluid aspirate cultures

C. XDR TB was not discovered in any lymph node aspirate culture

D. There was 100% concordance between the growth of XDR TB in sputum and pleural fluid cultures

### Activity Evaluation

**Table:** 

1. The activity supported the learning objectives.				
Strongly Disagree				Strongly Agree
1	2	3	4	5
2. The material was organized clearly for learning to occur.				
Strongly Disagree				Strongly Agree
1	2	3	4	5
3. The content learned from this activity will impact my practice.				
Strongly Disagree				Strongly Agree
1	2	3	4	5
4. The activity was presented objectively and free of commercial bias.				
Strongly Disagree				Strongly Agree
1	2	3	4	5
